# Levels of Soluble Endothelial Protein C Receptor Are Associated with CD4^+^ Changes in Maraviroc-Treated HIV-Infected Patients

**DOI:** 10.1371/journal.pone.0037032

**Published:** 2012-06-08

**Authors:** Silvia Nozza, Manuela Pogliaghi, Stefania Chiappetta, Vincenzo Spagnuolo, Gessica Fontana, Cristina Razzari, Giuseppe Tambussi, Elena Maria Faioni

**Affiliations:** 1 Division of Immunology Transplant and Infectious Diseases, Department of Infectious and Tropical Diseases, San Raffaele Scientific Institute, Milan, Italy; 2 Dipartimento di Medicina, Chirurgia e Odontoiatria, Università degli Studi di Milano e Medicina 3, Azienda Ospedaliera San Paolo-Polo Universitario, Milan, Italy; University of Cape Town, South Africa

## Abstract

**Background:**

Inflammation is a key feature of HIV infection and is correlated with long-term negative cardiovascular outcomes. Therapy-induced increases in CD4^+^ cell counts can control inflammation, as shown by decreases of coagulation and inflammation markers during efficacious therapy. Maraviroc, a CCR5-antagonist, has resulted in larger increases in CD4^+^ counts both in naïve and experienced subjects compared to traditional antiretroviral therapy.

**Objectives and Methods:**

To examine if a member of the protein C anticoagulant and anti-inflammatory pathway, and marker of coagulation and inflammation, the soluble endothelial protein C receptor, is modified by infection and therapy-related variables in patients treated with Maraviroc. Endothelial protein C receptor, together with other established markers of inflammation and coagulation (CRP, IL-6, D-dimer and soluble thrombomodulin) was studied in 43 patients on traditional antiretroviral therapy and in 45 on Maraviroc during 48 weeks of follow-up.

**Results:**

Soluble endothelial protein C receptor was the only marker that could discriminate at least partially between patients with a good response to Maraviroc and patients who did not respond with an adequate increase in CD4^+^ cell counts (more than 500 cells/µL by week 48).

**Conclusions:**

Elevated levels of soluble endothelial protein C receptor, a sensitive marker of endothelial damage, indicated a low level of inflammation and coagulation activation in Maraviroc treated patients not picked up by other widely used markers. Persistent elevated levels of this marker at 48 weeks from beginning of treatment with Maraviroc were related to a poor increase in CD4^+^ cells.

## Introduction

Persistent chronic inflammation is a key feature of HIV infection and it has many consequences, including an increased risk of cardiovascular events, compounded by other risk factors peculiar to HIV patients, such as certain therapeutic agents and lifestyle factors [1], [2]. Similarly to non infected patients, chronic inflammation as measured by biological markers, increases the likelihood of ischemic heart disease, approximately twofold in HIV+ patients. Infection by HIV was shown to induce oxidative stress and nitric oxide depletion on the endothelium and to impair endothelium dependent vasodilation [3], [4]. Low CD4^+^ cell counts were independently associated with an increased prevalence of carotid lesions [5]. Elevated levels of the pro-inflammatory cytokine interleukin-6, the fibrinolysis marker D-dimer and the acute phase protein CRP were associated with increased cardiovascular and all-cause-mortality in the SMART study, a large treatment interruption trial [6]. Moreover, in another treatment interruption trial, several inflammatory markers were found to be suppressed during treatment, and to rise during treatment interruption and the opposite was true for the anti-inflammatory biomarkers interleukin-10 and adiponectin [7].

The understanding of the regulation of the inflammatory response in stable HIV^+^ patients and of the effect of the different therapeutic agents on inflammatory markers is key to preventing cardiovascular disease and to identifying appropriate markers potentially predictive of cardiovascular disease and/or of response to the anti-inflammatory effect of therapy. Since inflammation and coagulation work together, it is reasonable to suppose that novel markers of regulatory coagulation pathways could be useful in this setting.

The protein C system is one of the main regulatory pathways of blood clotting: activated protein C inhibits two cofactors of the coagulation cascade (activated factors V and VIII) thereby limiting thrombin formation [8]. Protein C is activated on the endothelial surface by thrombin bound to the receptor thrombomodulin and aided by the endothelial protein C receptor (EPCR). Endothelial perturbation, especially by inflammation, negatively affects protein C activation by down-regulating the protein C pathway by several mechanisms [8]. Both thrombomodulin and EPCR are shed from the endothelial membrane during inflammation and can be measured in blood as soluble products [9], [10].

Treatment with CCR5-antagonist Maraviroc (MVC) has resulted in larger increases in CD4+ counts both in naïve [11] and experienced subjects [12]. Naïve patients treated with MVC had earlier decreases in markers of immune activation and inflammation, correlated with increased CD4^+^ cells [13]. In this study a small cohort of HIV patients treated with MVC was studied and compared over time with patients treated with traditional therapy to gain a preliminary insight into the usefulness of a panel of markers of inflammation, hemostasis and endothelial function including markers of the protein C pathway. We show that soluble EPCR (sEPCR) is a potentially useful marker of persistent inflammation and treatment failure in patients treated with MVC and that it is more sensitive than other traditional markers of inflammation and clotting.

## Methods

### Patients

Eighty-eight HIV-infected patients were enrolled in the study after providing written informed consent approved by San Raffaele ethics committee. There were 70 men and 18 women, median age was 49 years (range). Forty-three were on antiretroviral therapy (ART) while 45 were on ART and were placed on MVC at the beginning of the study (defined as baseline), according to screening genotype, previous resistance tests and viral tropism determined by the phenotypic test Trofile^®^. Median time from diagnosis at baseline was 16 years (range 5–23).

Blood sampling as well as a medical visit were performed at baseline and at 4, 12, 24, 36 and 48 weeks after starting therapy. Blood for coagulation and inflammation markers was collected in trisodium EDTA, centrifuged at 1400 g for 15 min and platelet poor plasma was separated and stored at −80°C. Plasma samples were rapidly melted in a 37°C water bath before testing. No refrozen samples were used.

The following variables were measured at each time point: blood glucose and insulin, lipid profile, and variables related to viral infection (CD4^+^ absolute count and percentage, CD4^+^/CD8^+^ ratio, HIV-RNA). Coagulation and inflammation markers [high sensitivity C-reactive protein (hsCRP), D-dimer (DD), interleukin-6 (IL-6), soluble thrombomodulin (sTM) and sEPCR] were measured up to week 24, except for sEPCR which was measured also at weeks 36 and 48.

At baseline, 24 and 48 weeks liver metabolism and HIV-DNA were evaluated as well.

## Methods

### Measurement of Biological Variables

Hs-CRP was measured by IMUCLONE CRP ELISA kit (American Diagnostica Inc, Stamford, CT, USA). Each assay contained a low level and a high level control. Inter-assay coefficient of variation (CV) for the high level was 8% and for the low level 16%. Normal reference range is: 0.2–10 µg/mL.

D-dimer was measured by IMUCLONE D-Dimer ELISA (American Diagnostica Inc, Stamford, CT, USA). Each assay contained a low level and a high level control. Inter-assay coefficient of variation (CV) for the high level was 5% and for the low level 21%. Normal values are <400 ng/mL (95^th^ percentile of the normal distribution).

IL-6 was measured by Immunoassay Kit, Human IL-6, Invitrogen Corporation, Camarillo, CA, USA. Since the kit did not provide a control, culture medium from inflamed, IL-6 producing primary smooth muscle cells was used as an internal control in each assay. The medium had been pre-tested in a different laboratory for IL-6 content. Inter-assay CV for this high level control was 24%. Normal reference range is: 720–12,000 pg/mL.

sTM was measured by ELISA Eli-pair, Cell Sciences, Canton, MA, USA. Normal plasma was used as an internal control (low level) and the inter-assay CV was 38%. Normal values are <44 ng/mL (95^th^ percentile of the normal distribution).

sEPCR was measured by a previously employed, home-made ELISA assay [14]. Briefly, microtiter plates (Nunc immuno plates MaxiSorp 96 wells, Sigma) were coated with 50 µL of 7 µg/mL JRK 1494, an anti-human EPCR mouse monoclonal antibody, in 15 mmol/L Na_2_CO_3_, 35 mmol/L NaHCO_3_, pH 9.6, at 4°C for 16 hours. The wells were washed five times with 20 mmol/L Tris-HCl, 0.15 mol/L NaCl, 0.05% (v/v) Tween 20, pH 7.5 (washing buffer), and were blocked with TBS-Casein (Bio-Rad Laboratories, 300 µL each well) for 4 hours at 37°C. The wells were then washed, 50 µL samples were added in duplicate wells, and the plates were incubated for 1 h. The wells were aspirated, washed five times with washing buffer, and 50 µL of 2,2 µg/mL biotinilated JRK 1495 anti-human EPCR mouse monoclonal antibody were added. The plates were incubated for 1 h, washed five times, and 50 µL streptavidin–conjugated HRP diluted 1∶3000 (Sigma) were added and incubated for one additional hour. The wells were washed five times, and the substrate SIGMA FAST OPD (o-phenylenediaminedihydrochloride, Sigma) was added. The color development was stopped with 3 M H_2_SO_4_, and the endpoint absorbance at 490 nm was read. Each plate contained standards in duplicate wells from 62.5 to 1000 ng/mL purified human soluble EPCR in washing buffer. The inter-assay CV was 7%.

Normal values are <297 ng/mL (95^th^ percentile of the normal distribution).

Mouse monoclonal antibodies against human EPCR JRK1494 and JRK1495, as well as human soluble EPCR, were a kind gift of C.T. Esmon (Cardiovascular Biology, Oklahoma Medical Research Foundation, Oklahoma City, OK, U.S.A.).

All other tests were part of good clinical practice protocols and were carried out at the central laboratory of the San Raffaele Scientific Institute.

### Statistical Analysis

Demographic, clinical and laboratory data were stored in an electronic database. Results for continuous variables are reported as median and range, for dichotomic variables as frequencies in percent or absolute counts. Differences between groups for dichotomic variables were evaluated by Fisher’s exact chi-square test. Differences by time and by treatment group in continuous variables were analysed first by analysis of variance for repeated measures, and then by direct comparison. P values <0.05 were considered significant.

## Results

At baseline, 43 patients with not CCR5-tropic virus started salvage antiretroviral therapy based on genotypic test: 4 (9%) with enfuvirtide, 10 (24%) with protease inhibitors and 29 (67%) with non nucleoside reverse transcriptase inhibitors; all patients received raltegravir. Forty-five with CCR5-tropic virus started therapy containing MVC: 8 (18%) at standard dosage (300 mg twice daily); 9 (20%) in association with protease inhibitors (150 mg twice daily); 28 (62%) in association with non nucleoside reverse transcriptase inhibitors (600 mg twice daily).

Patients on ART and on ART with MVC (i.e. at start of MVC therapy) differed for triglycerides and direct bilirubin levels only, while age and all other laboratory variables were similar (Table 1). Moreover, patients did not differ at baseline based on positivity for HCV-antibodies, HBsAg or for the prevalence of hypertension. Patients in stage C were 28/43 (65%) in ART and 24/45 (53%) in ART with MVC (no significant difference between treatment groups). There were proportionally more men than women in the ART with MVC group compared to the ART only one (40 men, 5 women compared to 30 men, 13 women, χ^2^ = 4.941, p<0.05).

**Table 1:** Age and laboratory variables at baseline of patients on ART (n = 43) or ART and MVC (n = 45).

Variable	ART	ART with MVC
Age (years)	47 (37–63)	46 (41–68)
CD4^+^ (/µL)	230 (4–653)	261 (19–911)
CD4p (%)	12.1 (0.4–30.3)	14.5 (2.4–37.2)
CD4/CD8 (ratio)	0.19 (0.01–0.89)	0.21 (0.04–0.9)
HIV-RNA (copies/µL)	15054 (99–711,230)	14513 (77–917,150)
HIV-DNA (copies/µL)	235 (9–7,834)	258 (9–5,858)
Total cholesterol (mg/dL)	179 (106–270)	176 (98–277)
HDL Cholesterol (mg/dL)	34 (21–170)	37 (17–77)
LDL Cholesterol (mg/dL)	98 (45–174)	103 (24–168)
Triglycerides (mg/dL)	214 (63–734)*	134 (57–497)*
Glucose (mg/dL)	84 (54–350)	85 (67–316)
Insulin	14 (5–77)	13 (2–227)
HOMA	3.14 (0.67–49.54)	2.35 (0.36–60.43)
AST	30 (9–108)	28 (17–123)
ALT	34 (9–175)	30 (11–135)
GGT	42 (6–251)	32 (11–444)
ALP	93 (38–426)	80 (17–519)
Total bilirubin	0.54 (0.21–2.37)	0.53 (0.21–0.98)
Direct bilirubin	0.21 (0.08–0.85)§	0.13 (0.07–0.34)§

Results are presented as median and range.

* p = 0.013 for direct comparison (Mann-Whitney).

§ p = 0.003 for direct comparison (Mann-Whitney).

**Figure 1:**
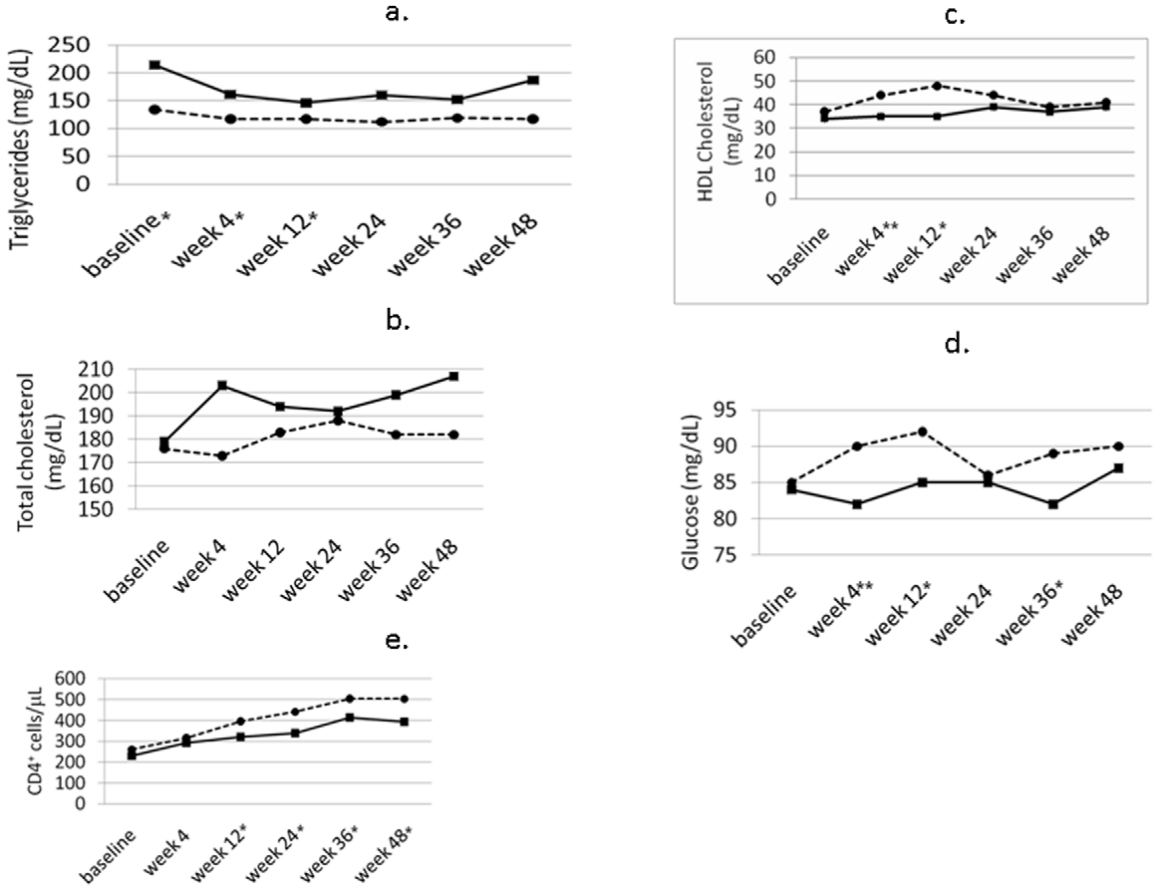
Changes in metabolic variables and CD4^+^ cell counts over time in patients on ART (solid line, squares) or ART with MVC (broken line, circles). * denotes p<0.05 and ** p<0.01 for differences between the two patient groups at the time shown (Mann-Whitney test after non parametric analysis of variance for repeated measures).

Levels of some variables changed over time and differed in the two groups. Figure 1 shows changes in metabolic variables and CD4^+^ cell counts over time in patients on ART and in patients who were administered MVC as well. Lipid profile was better in the ART with MVC group, and improved or remained stable over time. Especially HDL cholesterol increased sooner in the ART with MVC group than in the ART only patients. As also shown in the figure, CD4^+^ cell counts increased in both groups (p<0.001 at 48 weeks compared to baseline in both groups), but increments were higher and occurred sooner in patients on MVC compared to patients on traditional ART. Other CD4^+^ related variables (CD4^+^ absolute count and percentage, CD4^+^/CD8^+^ ratio) varied similarly to CD4^+^ cell counts, but did not show significant differences between the two treatment groups at any time (not shown). HIV-RNA decreased in both groups to undetectable levels in the majority of patients by week 4 and remained low through week 48 in both groups (Table 2).

**Table 2:** HIV-RNA copy levels by time and treatment group.

Time	ART		ART with MVC	
	HIV-RNA (Copies/mL)	N° of patientsundetectable (%)	HIV-RNA (Copies/mL)	N° of patients undetectable (%)
Baseline	15054 (99–711230)	0/43 (0)	14513 (77–917150)	0/45 (0)
Week 4	49 (49–8785)	27/42 (64)	49 (49–1435)	26/45 (58)
Week 12	49 (49–414678)	33/42 (79)	49 (49–51439)	34/45 (76)
Week 24	49 (49–173753)	35/42 (83)	49 (49–32935)	38/45 (84)
Week 36	49 (49–79798)	32/37 (86)	49 (49–314311)	35/42 (83)
Week 48	49 (49–623999)	33/35 (94)	49 (49–71702)	39/43 (91)

Data is presented as median and range. 49 copies is the lower limit of detection of the assay, and it was written in the database to indicate that the HIV-RNA was undetectable.

An interim analysis of coagulation and inflammation markers in the first 24 weeks showed no significant changes by time or by treatment group in hsCRP, IL-6, D-dimer or sTM (not shown). In general, median levels were within the normal range for CRP, IL-6 and sTM in both groups, while median D-dimer levels were increased above normal in both groups at all times. In contrast, since sEPCR levels showed a trend to change by time and treatment group, levels were measured beyond week 24 and are reported in Table 3.

**Table 3:** Median (range) levels of sEPCR.

Time	ART	ART with MVC
	sEPCR (ng/mL)	
Baseline	128 (71–576)	152 (81–400)
Week 4	131 (80–432)	144 (81–348)
Week 12	152 (84–504)	135 (73–432)
Week 24	138 (78–400)	127 (78–448)
Week 36	178 (108–496)	200 (132–456)
Week 48	186 (81–600)	189 (93–520)

**Figure 2:**
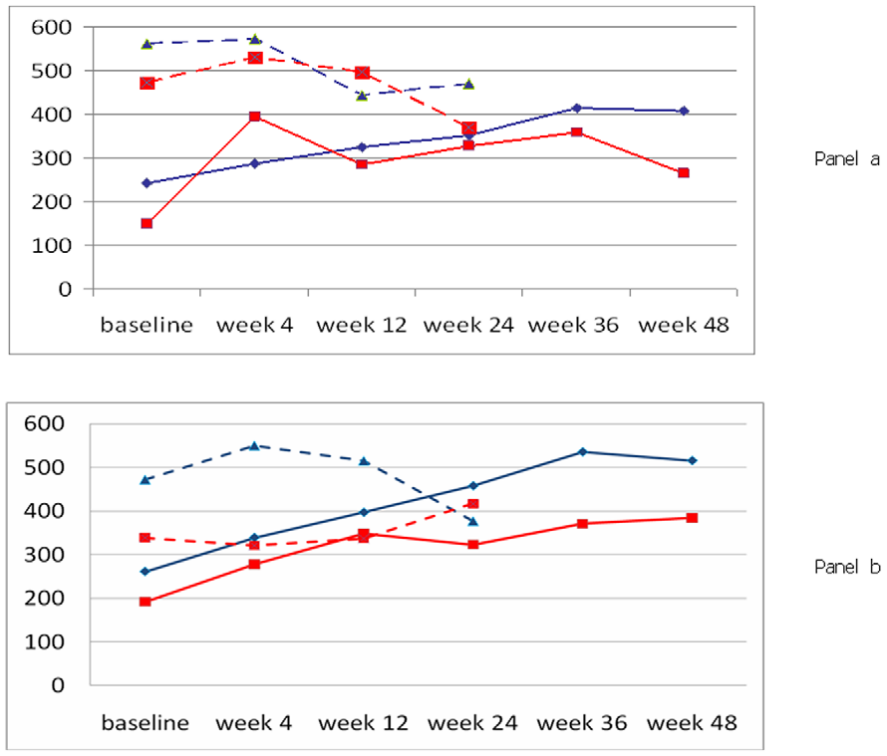
CD4^+^ cell counts (solid line) and D-dimer levels (broken line) in ART treated (panel a) or ART with MVC treated (panel b) patients, by sEPCR levels at week 48 (high levels = red lines, low levels = blue lines). High or low sEPCR levels were defined as sEPCR ≥ or <300 ng/mL (corresponding to the 95^th^ percentile of the normal distribution). As specified in the text, D-dimers levels were measured in all patients up to week 24, while sEPCR was measured in all patients up to weel 48.

sEPCR increased in both treatment groups, and levels at week 36 and 48 were higher than at baseline (p<0.001 for both treatment groups and both times). There were no differences between the two groups at any time, however more patients had levels above the reference range over the study time in the ART only (n = 26) compared to the ART with MVC treatment group (n = 18). If levels of soluble EPCR at week 48 above or below the 95^th^ percentile of the normal range were considered and CD4^+^ cell numbers were plotted separately for ART patients or ART with MVC, two different graphs were obtained (Figure 2). In both panels of Figure 2, CD4^+^ cells are observed to increase, as mentioned previously, and increases are overall greater in ART with MVC treated patients than in patients treated with ART only, also as reported above. However, it is interesting to note that in a group of ART with MVC treated patients who do not respond adequately to therapy, i.e. whose median increase in CD4^+^ cells at week 48 is less than 500 cells/µL, median levels of sEPCR at week 48 are high (2b). Also in ART treated patients levels of sEPCR are higher in patients who respond less, though both curves are persistently below the arbitrary threshold of 500 cells/µL (Figure 2a). Finally, D-dimer levels are also shown in Figure 2 (dotted lines): differences are observed in D-dimer levels in patients with high or low sEPCR levels at week 48. D-dimer levels changes are apparently inversely related to CD4^+^ cell counts changes, especially in MVC treated patients, though numbers were too small for statistical analysis.

## Discussion

In this study we observed that treatment experienced patients with a CCR5-tropic HIV1 virus treated with MVC in addition to ART showed a better lipid profile regardless of associated treatment and a greater and more precocious increase in CD4^+^ cell counts over time compared to patients on ART alone, while the decrease in viral load was comparable (by week 4 more than 50% and by week 48 more than 90% of patients had undetectable levels of HIV mRNA in both groups). In naïve patients, MVC improved lipid profiles [15]. Observed CD4^+^ cell counts increases are similar to those reported in previous larger studies [11], [12]. Median CD4^+^ cell counts in the ART with MVC group were 500 cells/µL by week 36 and then leveled off.

Inflammation and coagulation markers were studied as well, since both antiretroviral therapy and HIV infection are known to induce an inflammatory response and coagulation activation as a consequence of endothelial perturbation [6], [7]. IL-6 and hsPCR were low in both groups, indicating that patients included in the study were apparently in a stable, non-inflamed phase of the disease. D-dimer levels, a marker of fibrinolysis, were not significantly different between the two treatment groups and were only moderately increased compared to normal. Soluble EPCR increased in both treatment groups over time. Interestingly, the levels of sEPCR (above or below the 95^th^ percentile of the normal distribution) partially discriminated between patients who achieved and patients who did not achieve 500 CD4^+^ cells/µL by week 48. In general, the slope of increase in CD4^+^ by high or low levels of sEPCR at week 48 was significantly different. In patients who responded to MVC an exponential increase in CD4^+^ cells was associated with low levels of sEPCR at week 48 and low levels of D-dimer, and the converse was true in patients who did not respond adequately to MVC. The same trend were observed also in patients on traditional ART, though less marked.

sEPCR is generated at the endothelial surface by cleavage of the extracellular portion of the receptor. Cleavage occurs through a metalloprotease [16], induced by endothelial activation by thrombin, IL-1, TNF-α, or endotoxin, i.e. by coagulation and/or inflammatory activation, though in vivo thrombin activation of the endothelium seems to play the major role. sEPCR was shown to be a very sensitive marker of endothelial dysfunction and coagulation: for example, sEPCR levels were increased in sepsis and lupus erythematosus, two conditions known to be associated with inflammation and coagulation, more than soluble thrombomodulin, an established marker of endothelial damage that belongs to the same anticoagulant pathway as EPCR [10]. In the current study, the increase in sEPCR levels in some patients on ART or ART with MVC treatment, even in the absence of an increase in IL-6 or hsPCR, suggests a selective low level activation of the endothelium by inflammation and coagulation. Moreover, sEPCR is more sensitive to this inflammatory state than soluble thrombomodulin which was found unchanged over time and treatment group.

Three important suggestions can be derived from this study. First, anti-HIV therapy seems to control inflammation and coagulation, and is associated with an increase in CD4^+^ cells. MVC increases faster and sooner the CD4^+^ cell count, but the same relationship between CD4^+^ cell counts, sEPCR and D-dimer can be observed also in ART treated patients, though it’s less clear-cut. Second, a low-grade inflammatory and hypercoagulable state is observed also in patients with stable long-lasting disease and low levels of other, more traditional inflammatory and coagulation markers. Third, some patients who do not respond to therapy, either ART or MVC, by an increase in CD4^+^ cell counts, independently of reduction in viral load, have a persistent or increasing inflammation and coagulation activation. Fundenburg and coworkers also reported that decreases in inflammatory and immune markers in HIV^+^ patients were associated with an increase in CD4^+^ cell counts.^13^ The authors also suggested that the early, modest differential effect of MVC on several markers compared to the comparator of the trial suggested an additional direct effect of the drug on immune activation and inflammation. Moreover, MVC was reported to decrease T cell activation and to modulate the immune activation present in HIV patients, though through which mechanism is not clear [17].

Elevated D-dimer was found to be strongly associated with mortality and also to predict cardiovascular disease in HIV+ patients [18]. No studies of sEPCR were performed in HIV^+^ patients, but sEPCR levels were shown to be associated with a hypercoagulable state and cardiovascular disease in diabetic patients [19] and autoantibodies to EPCR were associated with increased risk of myocardial infarction in women [20]. Coagulation and inflammation are tightly linked and mutually enhancing, thus strategies that control inflammation also are likely to control coagulation activation. Furthermore, EPCR per se was shown to possess multiple anti-inflammatory activities, so that shedding of EPCR from the endothelium contributes to the loss of both anticoagulant and anti-inflammatory potential, generating a vicious cycle. Therapies, such as MVC, that inhibit viral entry in the CD4^+^ cells and increase their numbers, have a potential to control coagulation activation and inflammation in responsive patients at least in the short term. In the setting of antiretroviral naïve patients, Maraviroc was not associated with elevations in lipids and showed beneficial effects on lipid profiles of dyslipidemic patients [21]. If confirmed in patients with higher inflammatory levels and in longer follow-up time, such a therapeutic strategy could potentially be effective also in preventing the cardiovascular consequences of HIV infection associated with chronic inflammation.

### 

#### Informed consent

All patients signed informed consent for participation of Maraviroc Expanded Access Program.
